# Supplementary material for: Modular prophage interactions driven by capsule serotype select for capsule loss under phage predation
Source: ISME J. 2020 Jul 30;14(12):2980–96. doi: 10.1038/s41396-020-0726-z (PMC7784688; doi:10.1038/s41396-020-0726-z)
Supplement: Supplementary file 1 — Supplemental Material [file 41396_2020_726_MOESM1_ESM.pdf]

SUPPLEMENTAL MATERIAL

Modular prophage interactions driven by capsule serotype select for capsule loss under phage predation

Jorge A. Moura de Sousa[1], Amandine Buffet[1], Matthieu Haudiquet[1,2], Eduardo P.C. Rocha[1,] & Olaya Rendueles*[1]

[1] Microbial Evolutionary Genomics, Institut Pasteur, CNRS, UMR3525, Paris, 75015, France,
[2] Ecole Doctoral FIRE – Programme Bettencourt, CRI, Paris, 75005, France

* olaya.rendueles-garcia@pasteur.fr

Table of Contents

SUPPLEMENTAL FIGURES 2

SUPPLEMENTAL TABLES 12

SUPPLEMENTAL REFERENCES 25

SUPPLEMENTAL FIGURES

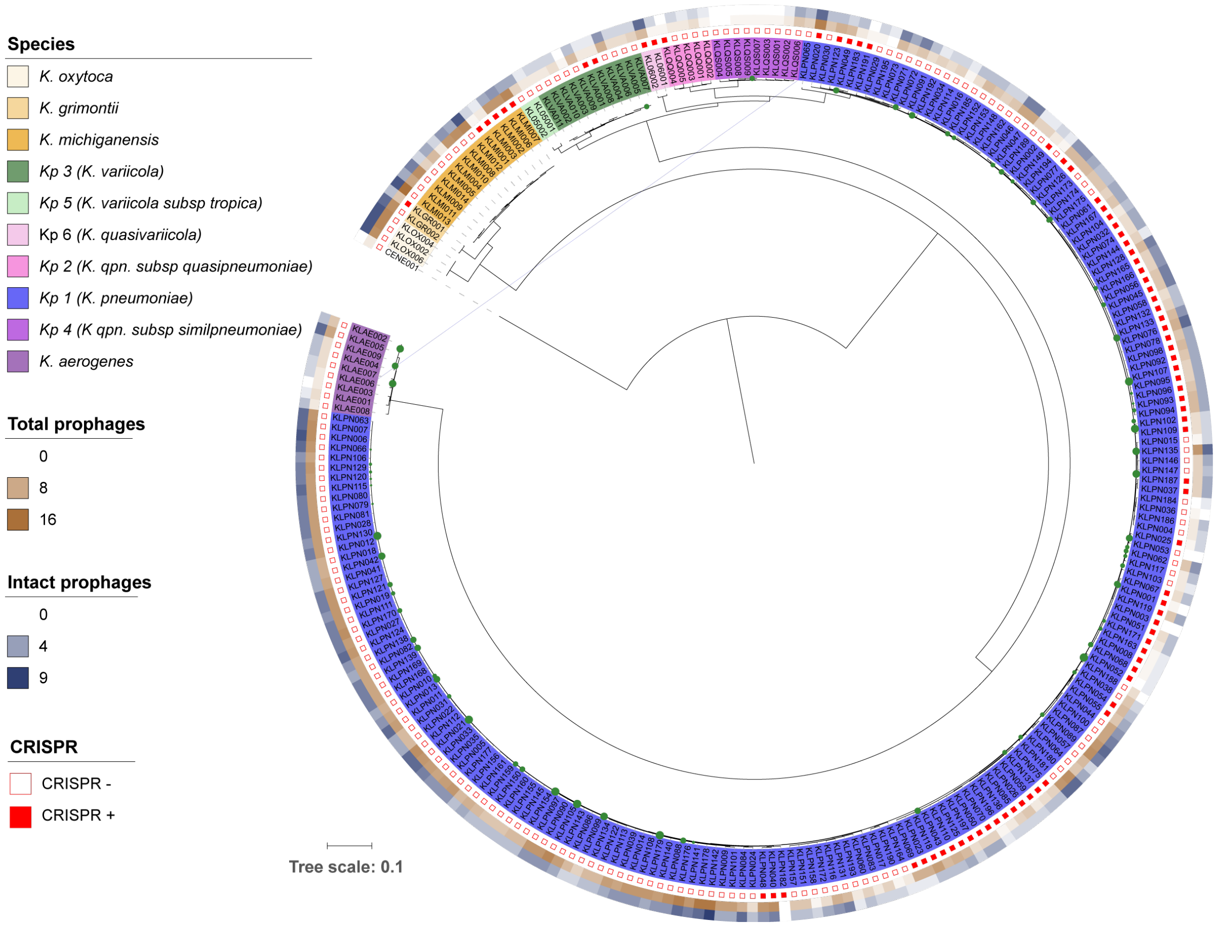

Figure S1. Phylogenetic tree of 254 Klebsiella strains analysed in this study. The tree was built using the protein sequences of the 1116 families of the core genome of *Klebsiella spp*. Red squares on the outer part of the tree indicate the presence (full) or absence (empty) of CRISPR-systems. The next two columns indicate the total number of prophages (brown) and the number of intact prophages (blue). Green circles indicate bootstrap values less than 99 (for clarity purposes). The size of the circle is proportional to the bootstrap value.

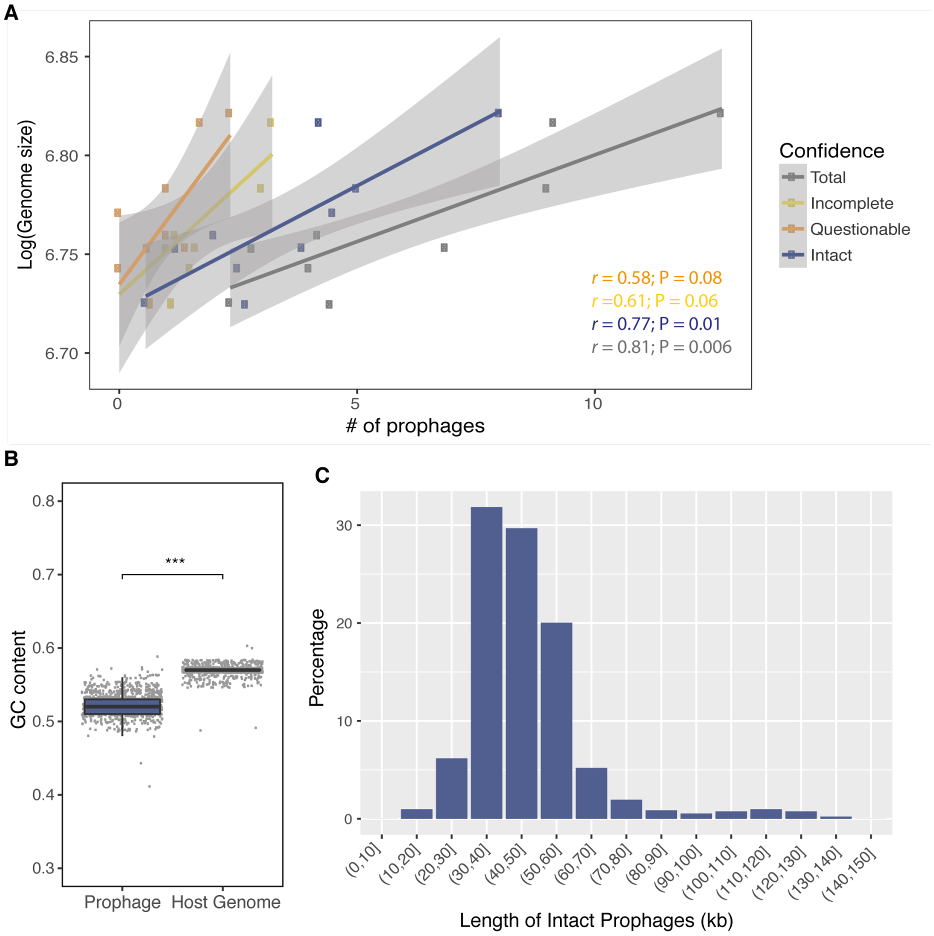

Figure S2. Characteristics of *Klebsiella spp* prophages. A. Correlation between the average number of prophages per genome per species with average genome size of the species. Each point represents the average of all strains of one species. P values correspond to Spearman's correlation. **B.** GC content of "intact" prophages and host genomes. Each dot represents individual genomes. *** P < 0.001, Wilcoxon test. **C.** Distribution of the length of "intact" prophages.

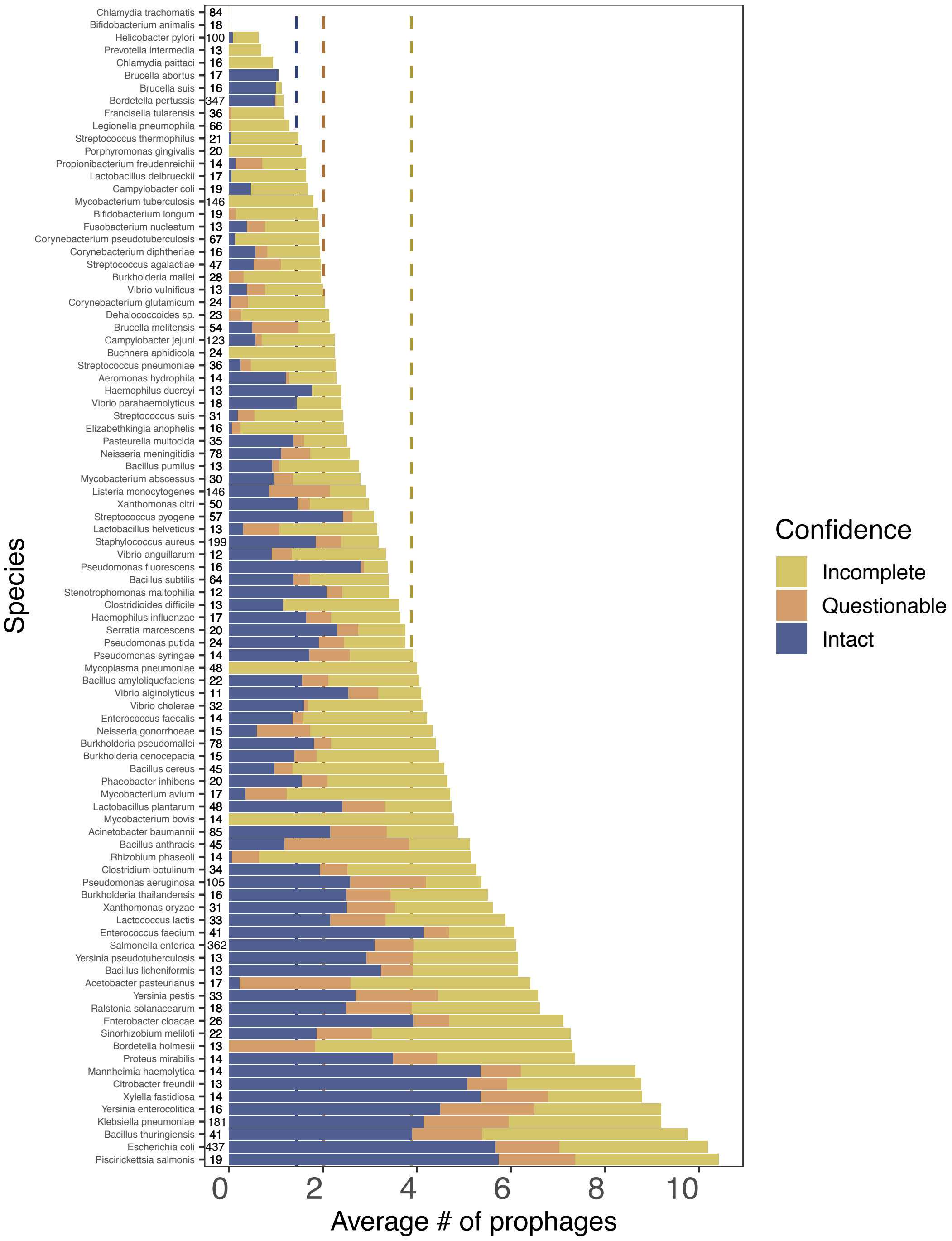

Figure S3. Average number of prophages in the 100 species with most genomes sequenced. All complete genomes were downloaded November 2016 from NCBI RefSeq (ftp://ftp.ncbi.nih.gov/genomes/)), regrouped by species, and the 100 most sequenced species were selected. Their genomes were analysed by PHASTER for phage detection. Digits indicate the number of analyzed genomes. Dashed line indicated average of each category for the dataset.

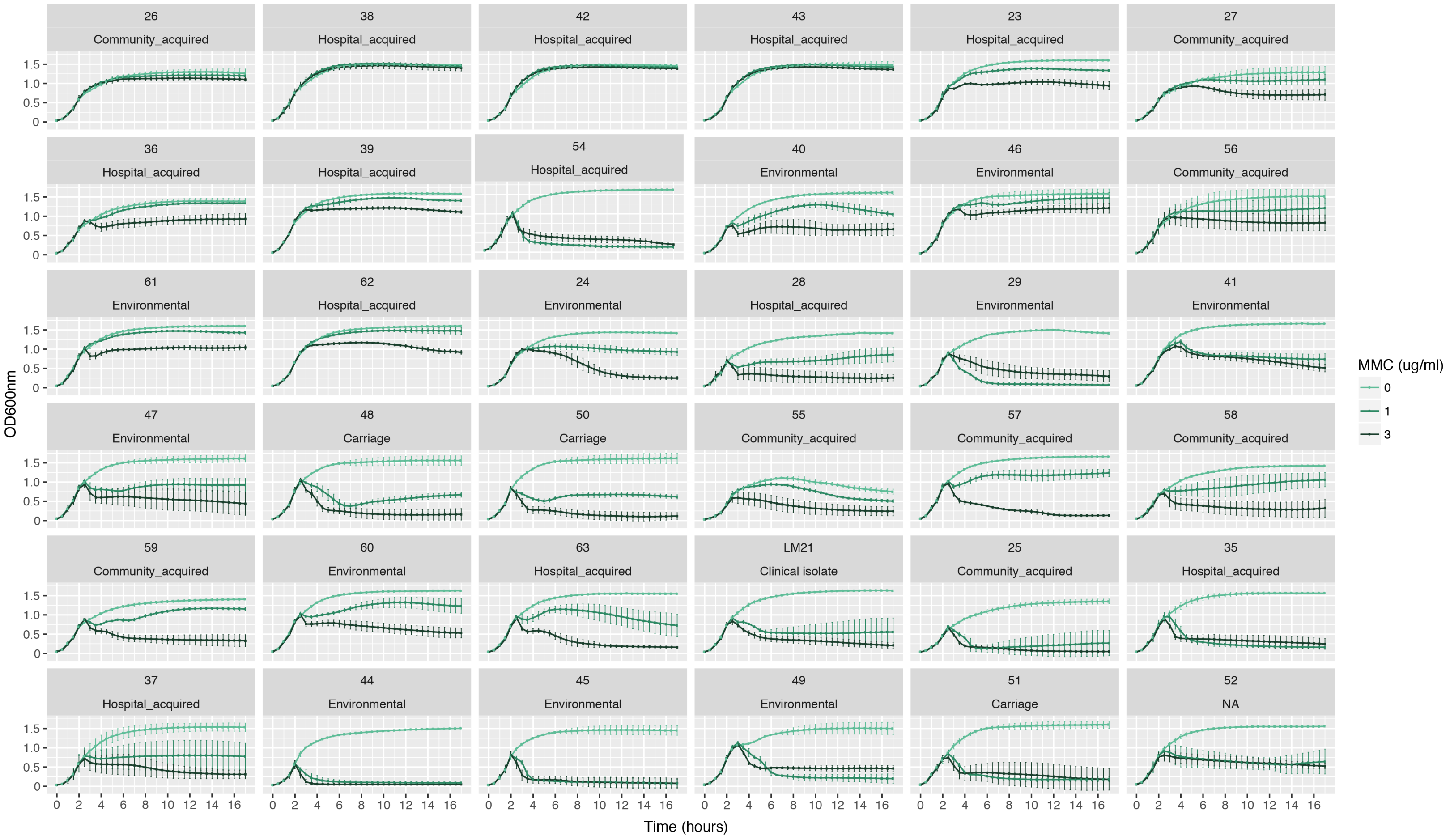

Figure S4. Growth of all 35 strains used in this study at different concentrations of mitomycin C (MMC). The ecological source of the isolates is also indicated.

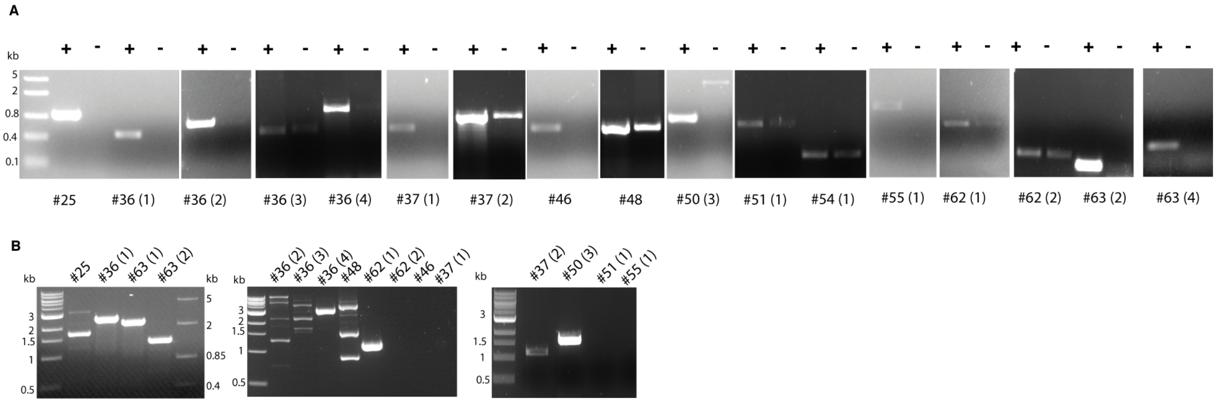

Figure S5. Excision of temperate phages from their bacterial host. A. PCR specific to different phages encoded in different strains, detected in induced supernatants (+) but also in non-induced (-). Filtered and PEG-precipitated supernatants were treated with double-stranded DNAse (Thermo Scientific) for 10 minutes at 37°, and then 20 minutes at 65°, for enzyme degradation. One μl was used as PCR matrix, and 5μl of PCR were loaded on an agarose gel. Numbers correspond to the host genomes, as displayed in Figure 3. The numbers in parenthesis identify the prophage in the genome. **B.** Recircularisation proof for several temperate phages. Primers were designed to match prophage regions at the borders, but in opposite genomic directions. Presence of a PCR product indicated that the phage has excised and recircularized.

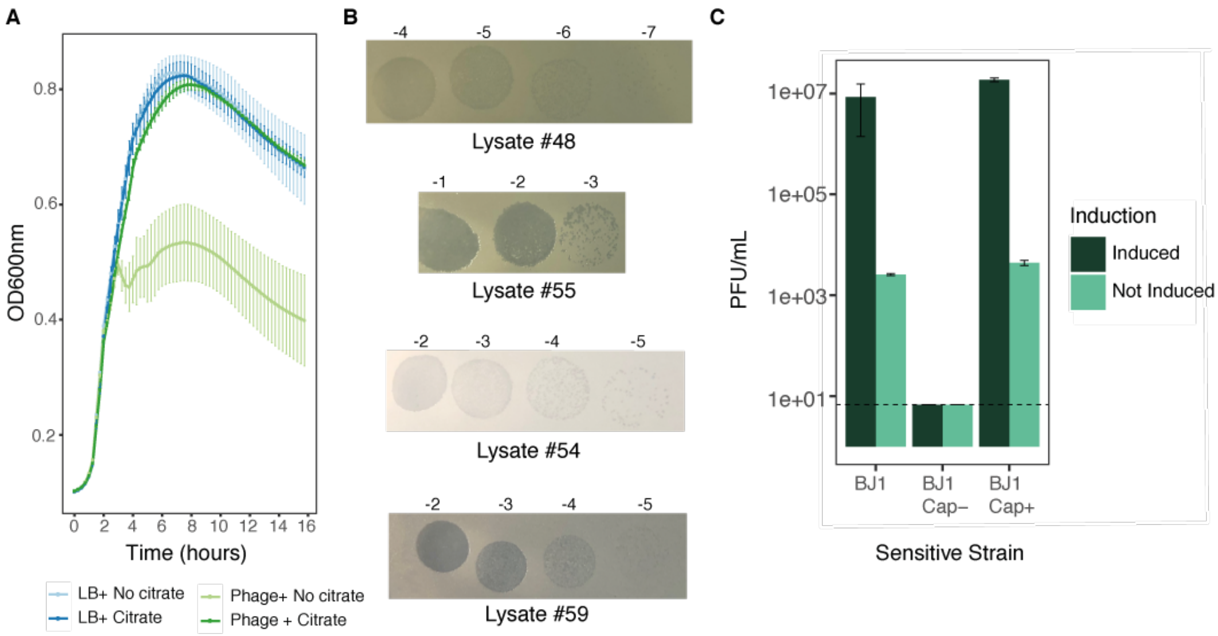

Figure S6. Prophages can excise and infect other *Klebsiella* strains. **A.** Growth of BJ1 strain after the addition of different concentrations of purified phage (shades of green) produced by strain #54 in the presence (full line) or absence (dashed line) of citrate (0.2%). **B.** Serial dilutions of lysates from different strains on an overlay of strain BJ1. Numbers indicate the dilution. **C.** PFU per ml produced by PEG-precipitation and filtered-supernatants lysates of strain #54 which were induced and not induced by MMC. Dashed line indicates the limit of detection of our assay. All experiments were performed in triplicate and error bars correspond to standard deviation of independent biological replicates.

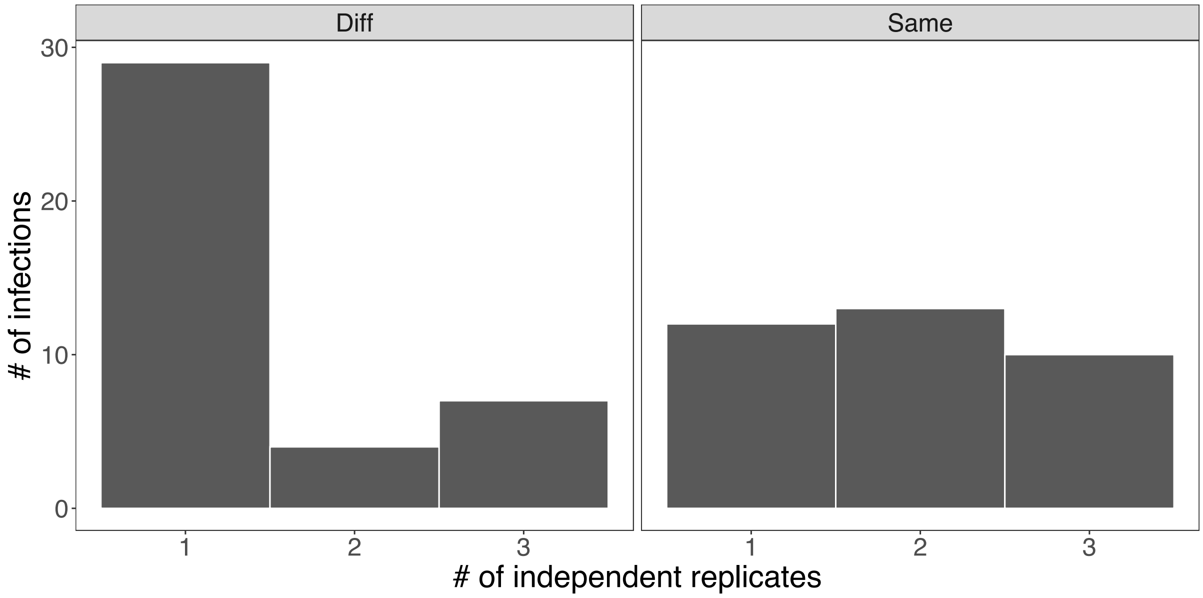

Figure S7. Number of independent replicates in which a given infection was observed. The infection matrix was built using three independent lysates over three independent overlays. In very few cases did the three independently-generated lysates infect (independent replicates). The graph reports the number of infections that were observed in one, two or three independent replicates. The panels are separated as to show infections from lysates from a bacteria with a different capsule locus type than that of the targeted bacteria (Diff, first panel) or from the same capsular locus type (Same, second panel).

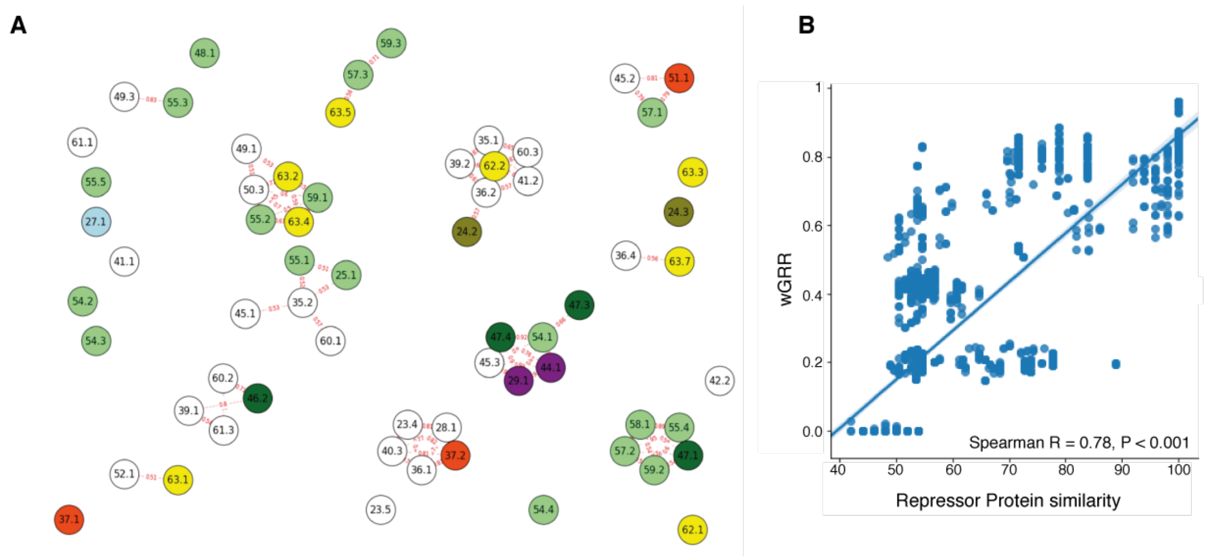

Figure S8. Phage similarity does not confer resistance to superinfection. A. Network of prophages (intact & questionable) of 35 *Klebsiella* strains as calculated by the wGRR (indicated in red along the connexions), with a cut-off of 0.5. Node colors represent different capsule locus types (as in Figure 4). **B.** Correlation between the wGRR of intact prophage genomes of the *Klebsiella* strains used in this study, and the protein sequence similarity of their respective phage repressors.

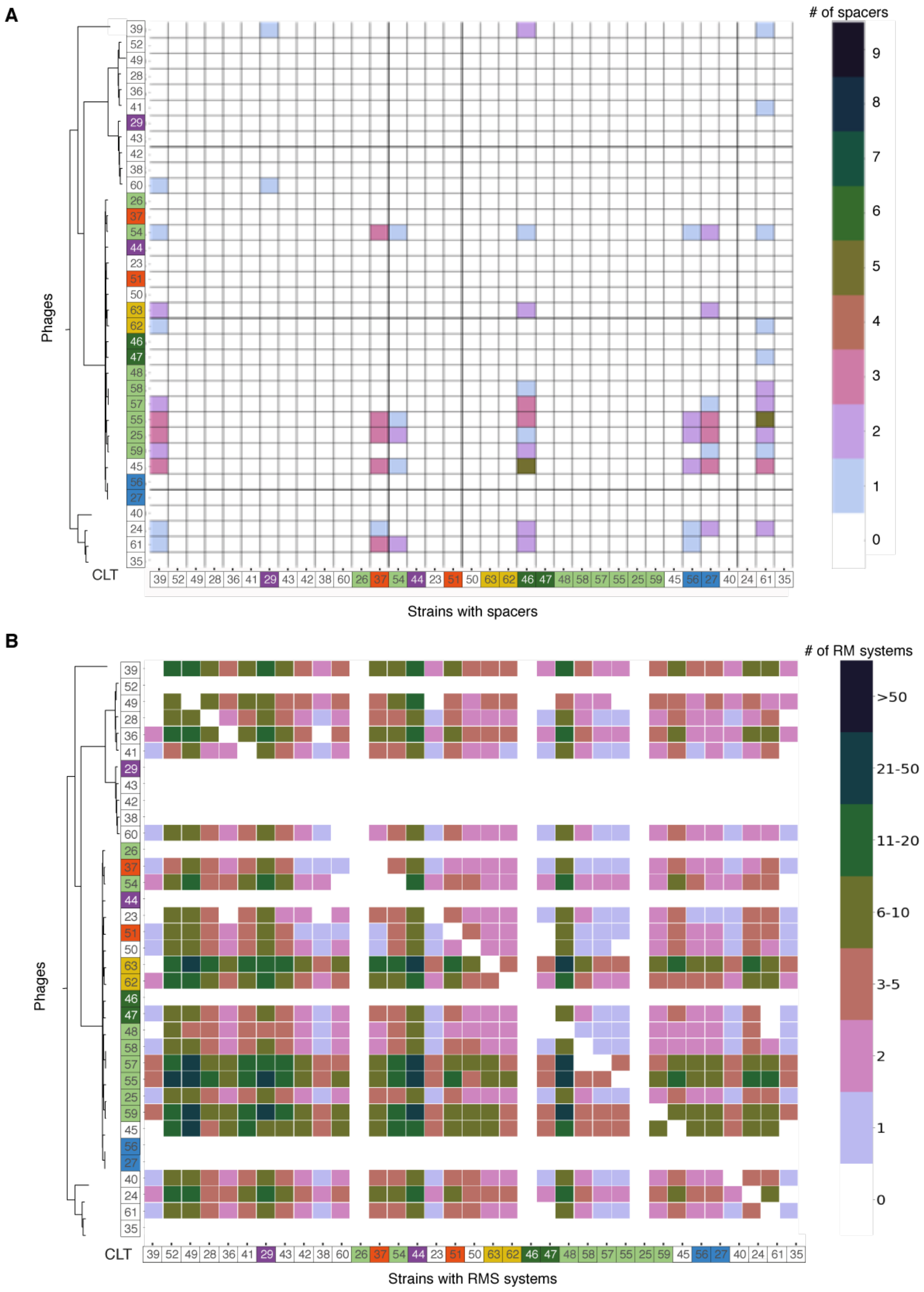

Figure S9. Number of spacers and R-M systems targeting intact prophages genomes from other strains. A. Spacer sequences of each CRISPR-Cas array were identified and blasted against the genomes of all intact prophages. The colours in the matrix reflect the number of different spacers in a strain (x-axis) that matches a prophage in a given strain (y-axis). **B.** The sequence motifs targeted by each R-M system present in one strain but absent in another were identified and blasted against the genomes of all intact prophages. The colours in the matrix reflect the number of R-M systems in a strain (x-axis) that matches a prophage hosted in another strain (y-axis).

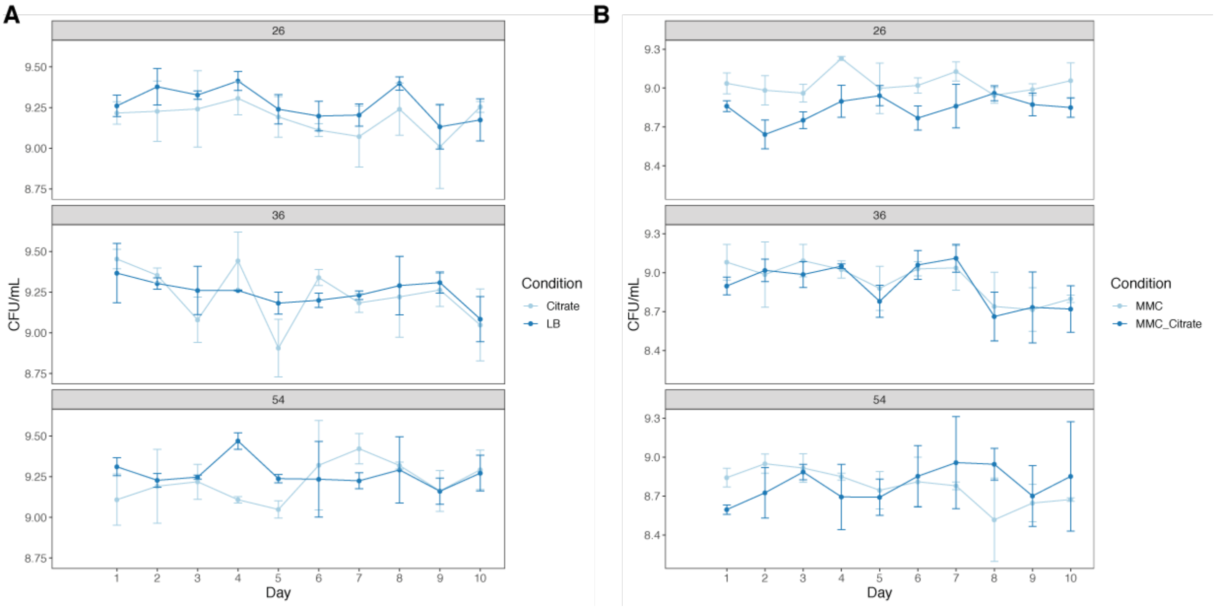

Figure S10. Number of CFU/mL per day, per strain and per condition. Numbers in grey banner indicate the strain. The area under the curve between the populations grown in the presence or absence of citrate (LB vs LB + citrate, and MMC vs MMC + citrate) was not significantly different for all strains (T-test, $P > 0.05$).

SUPPLEMENTAL TABLES

Table S1. Colicins and microcins identified in the strains experimentally tested in this study. Indicated in bold are the profiles with a match higher than 55%.

Strain #	Contig #	Name	Start	End	Strand	Function	Match (%)	E-value	UniProt Profile	Strain infectivity
63	4	orf00041	15639	15788	+	Lysis protein	**91.84**	2.00E-25	P02987	Halo on 19 different strains
63	4	orf00042	15297	15554	+	Cloacin immunity protein	**98.82**	5.00E-55	IMMC_ECOLX	
63	4	Colicin_E7	13602	15287	+	Colicin_E7	**71.02**	1.00E-79	Q47112	
23	2	Colicin	1465	3786	+	Colicin	48.85	9.00E-40		Mild halo on overlay of strain #47
23	2	PyocinIm	3788	4066	+	Colicin-E9 immunity protein	50.62	2.00E-22	IMM9_ECOLX	
23	3	Colicin	1465	3786	+	Colicin	48.85	9.00E-40		Mild halo on overlay of strain #47
23	3	PyocinIm	3788	4066	+	Colicin-E9 immunity protein	50.62	2.00E-22	IMM9_ECOLX	
48	59	PyocinIm	2499	2756	+	Colicin-E9 immunity protein	50	6.00E-23	IMM9_ECOLX	Halo on 8 strains, 6 of which have the same CLT
48	59	Colicin	2015	2497	+	Colicin	49.24	5.00E-43		
44	127	PyocinIm	5265	5522	+	Colicin-E9 immunity protein	50.62	6.00E-23	IMM9_ECOLX	No inhibition halo
44	127	orf00039	5093	5263	+	Colicin-E7	**55.36**	1.00E-13	Q47112	
44	127	orf00042	4369	4980	+	Colicin-D	33.72	2.00E-14	CEAD_ECOLX	
27	281	Microcin_M	14588	14767	-	Bacteriocin_Iic	87.23	8.00E-27		No inhibition halo
27	281	LanT	15041	17113	-	Probable microcin-H47 secretion/processing ATP-binding protein mchF	93.19	0	MCHF_ECOLX	
27	281	HlyD	17130	18191	-	Microcin H47 secretion protein mchE	92.07	0	MCHE_ECOLX	
27	281	MicD	18530	19021	-	RTX-II toxin-activating lysine-acyltransferase ApxIIC	34.57	9.00E-07	P0A3I4	
27	281	orf00052	21559	21828	+	Bacteriocin_Iic			PF10439	
27	281	Microcin_I47	22705	22872	+	Microcin_I47	50	4.00E-11		
27	281	orf00067	25962	26249	+	Microcin E492 immunity protein	**100**	5.00E-61	IM92_KLEPN	
27	281	Microcin_24	26329	26535	+	Microcin_E492147.2	**100**	2.00E-41		
27	281	ABC	34456	36174	-	Putative multidrug export ATP-binding/permease protein	33.4	3.00E-83	P71082	

27	281	LanT	36245	36469	-	ABC-type bacteriocin/lantibiotic exporters, contain an N-terminal double-glycine peptidase domain	33.4	4.00E-11	Q2S9A9_HAHCH	
36	2	PyocinIm	97234	97491	+	Colicin-E9 immunity protein	50.62	7.00E-23	IMM9_ECOLX	Inhibition halo on #38 &#40. Lysogenization of #38 (Figure 4D)
36	2	Colicin	95214	97232	+	Colicin	48.85	3.00E-40		
28	76	Colicin_E7	3933	5618	+	Colicin_E7	**71.02**	1.00E-79	Q47112	Inhibition halo on #60, and mild halo on #59 and #61
28	76	orf00023	5628	5885	+	Cloacin immunity protein	**98.82**	5.00E-55	IMMC_ECOLX	
28	76	orf00024	5970	6119	+	Lysis protein	**91.84**	2.00E-25	LYS0_ECOLX	
52	89	orf00019	1619	1774	+	Lysis protein for colicin A	60.78	8.00E-14	LYS1_CITFR	
52	89	PyocinIm	1254	1511	+	Colicin-E9 immunity protein	50	6.00E-23	IMM9_ECOLX	
52	89	Colicin	632	1252	+	Colicin	49.24	3.00E-42		
42	25	Colicin	1474	3414	+	Colicin	48.85	2.00E-40		Mild halo on overlay of strain #47
42	25	PyocinIm	3416	3694	+	Colicin-E9 immunity protein	50.62	2.00E-22	IMM9_ECOLX	
43	40	Colicin	4914	5807	+	Colicin	49.24	2.00E-41		Mild halo on overlay of strain #47
43	40	PyocinIm	5809	6066	+	Colicin-E9 immunity protein		6.00E-23	IMM9_ECOLX	
29	168	PyocinIm	3503	3760	+	Colicin-E9 immunity protein		7.00E-23	IMM9_ECOLX	Mild halo on overlay of strain #52, #60 and #62
29	168	orf00024	2382	2564	-					
29	168	Colicin	2608	3501	+	Colicin	49.24	2.00E-41		
39	3	PyocinIm	77904	78161	+	Colicin-E9 immunity protein	50.62	6.00E-23	IMM9_ECOLX	Mild halo on overlay of strain #47
39	3	orf00035	76783	76965	-					
39	3	Colicin	77009	77902	+	Colicin	49.24	2.00E-41		
26	1	Microcin_24	3768943	3769149	-	Microcin_E492147.2	**100**	2.00E-41		Mild halo on overlay of strain #47
26	1	orf00033	3769229	3769516	-	Immunity Microcin	**100**	5.00E-61	IM92_KLEPN	
26	1	Microcin_I47	3772606	3772773	-	MccI47_mchS21	49.2	3.00E-14		
26	1	orf00048	3773650	3773919	-	Bacteriocin_Iic				
26	1	MicD	3776457	3776948	+	RTX-II toxin-activating lysine-acyltransferase ApxIIC	34.57	9.00E-07	P0A3I4	
26	1	HlyD	3777575	3778348	+	Microcin H47 secretion protein mchE	**93**	8.00E-174	MCHE_ECOLX	
26	1	LanT	3778365	3780437	+	Probable microcin-H47 secretion/processing ATP-binding protein mchF	**93.19**	0	MCHF_ECOLX	
26	1	Microcin_M	3780711	3780890	+	Bacteriocin_Iic	**87.23**	8.00E-27		

Table S2. HMM profiles and sequences used to detect depolymerases. HMM profiles associated with bacteriophage-encoded depolymerases from multiple bacterial species as well as the genetic sequences of five experimentally validated depolymerases.

Enzyme class	Polymer	Profile	Predicted domains	Cut-offs
Sialidases	Sialidic acid	PF12218.6	End_N_terminal	e-value maximum 1e-3, minimum profile coverage 30%
		PF12217.6	End_beta_propel	
		PF12219.6	End_tail_spike	
		PF13884.4	Peptidase_S74	
		PF11962.6	Peptidase_G2	
Levanases	Levan	PF00251.18	Glyco_hydro_32N	
		PF08244.10	Glyco_hydro_32C	
Xylosidases	Xylan	PF01229.15	Glyco_hydro_39	
Dextranases	Dextran	PF13199.4	Glyco_hydro_66	
Peptidases	Poly-y-glutamate	PF05908.9	DUF867	
Hyaluronidases	Hyaluronate	PF07212.9	Hyaluronidase_1	
Pectin/pectate lysases	Galacturonate	PF00544.17	Pec_lyase_C	
		PF12708.5	Pectate_lyase_3	
Lipases	Triacylglycerols	PF14606.4	Lipase_GDSL_3	

Sequence	Description	Cut-offs	Reference
YP_009226010.1	Tail fiber protein	e-value maximum 1e-5, minimum coverage 40%, minimum identity 40%	(Majkowska-Skrobek et al 2016)
BAP15746.1	Hypothetical protein		(Lin et al 2014)
BAQ02780.1	Tail fiber		(Pan et al 2015)
S1-1	Tail spike		(Pan et al 2017)
S2-6	Tail spike		

Table S3. Prophage proteins matching an HMM profile for capsule depolymerases. HMM profiles are described in Table S2.

Phage protein hit	Strain #	Host CLT	Profile	E-value best domain	Coverage (%) best domain
KLAE.0918.00009.4_00069		KL107	Pectate_lyase_3	1.30E-13	77.21
KLOX.0918.00009.8_00003		KL68	Pectate_lyase_3	3.40E-11	85.58
KLAE.0918.00005.3_00031		KL68	Peptidase_S74	3.30E-11	98.28
KLAE.0918.00007.7_00003		KL68	Peptidase_S74	1.40E-09	93.10
KLAE.0918.00009.3_00003		KL107	Peptidase_S74	1.40E-09	93.10
KLMI.0918.00001.2_00027		KL152	Peptidase_S74	0.0002	91.38
KLMI.0918.00007.3_00067		KL43	Peptidase_S74	1.80E-07	91.38
KLOX.0918.00004.11_00064		KL18	Peptidase_S74	5.20E-08	96.55
KLOX.0918.00005.11_00073		KL68	Peptidase_S74	7.00E-11	96.55
KLOX.0918.00005.8_00051		KL68	Peptidase_S74	5.10E-10	93.10
KLOX.0918.00006.13_00056		KL68	Peptidase_S74	7.00E-11	96.55
KLOX.0918.00006.8_00051		KL68	Peptidase_S74	5.10E-10	93.10
KLOX.0918.00007.12_00003		KL68	Peptidase_S74	7.00E-11	96.55
KLOX.0918.00007.5_00008		KL68	Peptidase_S74	5.10E-10	93.10
KLOX.0918.00008.11_00022		KL43	Peptidase_S74	2.70E-10	96.55
KLOX.0918.00009.13_00004		KL68	Peptidase_S74	5.10E-10	93.10
KLOX.0918.00010.10_00051		KL70	Peptidase_S74	1.80E-07	91.38
KLOX.0918.00010.2_00054		KL70	Peptidase_S74	5.10E-10	93.10
KLPN.0918.00002.2_00001		KL1	Peptidase_S74	0.0002	91.38
KLPN.0918.00005.2_00004		KL38	Peptidase_S74	1.70E-07	94.83
KLPN.0918.00009.3_00027		KL67	Peptidase_S74	6.00E-05	93.10
KLPN.0918.00009.5_00002		KL67	Peptidase_S74	0.00012	93.10
KLPN.0918.00012.2_00002		KL1	Peptidase_S74	0.0002	91.38
KLPN.0918.00017.3_00001		KL47	Peptidase_S74	5.20E-07	96.55
KLPN.0918.00018.5_00001		KL47	Peptidase_S74	5.20E-07	96.55
KLPN.0918.00020.1_00030		KL1	Peptidase_S74	0.0002	91.38
KLPN.0918.00028.1_00029		KL1	Peptidase_S74	0.0002	91.38
KLPN.0918.00032.1_00029		KL1	Peptidase_S74	0.0002	91.38
KLPN.0918.00033.2_00029		KL47	Peptidase_S74	5.20E-07	96.55
KLPN.0918.00036.3_00002		KL1	Peptidase_S74	0.0002	91.38
KLPN.0918.00038.3_00025	#25	KL2	Peptidase_S74	4.80E-07	96.55
KLPN.0918.00040.2_00002		KL30	Peptidase_S74	4.50E-07	91.38
KLPN.0918.00045.3_00025		KL2	Peptidase_S74	4.80E-07	96.55
KLPN.0918.00047.6_00017		KL17	Peptidase_S74	5.20E-07	96.55
KLPN.0918.00049.3_00001		KL2	Peptidase_S74	4.30E-07	96.55
KLPN.0918.00050.2_00001		KL30	Peptidase_S74	2.10E-06	93.10
KLPN.0918.00050.7_00070		KL30	Peptidase_S74	3.90E-09	93.10

KLPN.0918.00051.4_00027		KL30	Peptidase_S74	2.10E-06	93.10
KLPN.0918.00051.6_00097		KL30	Peptidase_S74	3.90E-09	93.10
KLPN.0918.00057.11_00066		KL19	Peptidase_S74	1.80E-07	91.38
KLPN.0918.00057.11_00071		KL19	Peptidase_S74	7.00E-08	91.38
KLPN.0918.00057.7_00020		KL19	Peptidase_S74	3.10E-10	96.55
KLPN.0918.00058.4_00002		KL21	Peptidase_S74	0.00012	93.10
KLPN.0918.00059.3_00002		KL14	Peptidase_S74	0.00012	93.10
KLPN.0918.00060.3_00002		KL14	Peptidase_S74	0.00012	93.10
KLPN.0918.00069.5_00003		KL107	Peptidase_S74	1.70E-06	93.10
KLPN.0918.00069.6_00002		KL107	Peptidase_S74	0.0002	91.38
KLPN.0918.00076.4_00012		KL27	Peptidase_S74	1.80E-07	91.38
KLPN.0918.00080.3_00051		KL64	Peptidase_S74	3.90E-09	93.10
KLPN.0918.00085.3_00030		KL2	Peptidase_S74	3.90E-09	93.10
KLPN.0918.00086.5_00080		KL64	Peptidase_S74	3.90E-09	93.10
KLPN.0918.00094.3_00002		KL1	Peptidase_S74	0.0002	91.38
KLPN.0918.00094.6_00003		KL1	Peptidase_S74	3.90E-09	93.10
KLPN.0918.00097.4_00079		KL2	Peptidase_S74	3.90E-09	93.10
KLPN.0918.00101.5_00021		KL64	Peptidase_S74	3.70E-05	91.38
KLPN.0918.00102.5_00067		KL64	Peptidase_S74	0.0002	91.38
KLPN.0918.00113.6_00053		KL51	Peptidase_S74	3.90E-09	93.10
KLPN.0918.00114.4_00017		KL64	Peptidase_S74	3.90E-09	93.10
KLPN.0918.00114.8_00083		KL64	Peptidase_S74	6.20E-07	91.38
KLPN.0918.00121.5_00011		KL64	Peptidase_S74	1.70E-07	91.38
KLPN.0918.00121.5_00017		KL64	Peptidase_S74	6.30E-07	91.38
KLPN.0918.00126.7_00008		KL64	Peptidase_S74	0.0002	91.38
KLPN.0918.00128.7_00075		KL105	Peptidase_S74	5.50E-07	96.55
KLPN.0918.00131.7_00001		KL64	Peptidase_S74	5.20E-07	96.55
KLPN.0918.00132.4_00002		KL21	Peptidase_S74	0.00012	93.10
KLPN.0918.00133.14_00039		KL47	Peptidase_S74	5.20E-07	96.55
KLPN.0918.00133.4_00039		KL47	Peptidase_S74	5.20E-07	96.55
KLPN.0918.00135.14_00039		KL47	Peptidase_S74	5.20E-07	96.55
KLPN.0918.00135.4_00039		KL47	Peptidase_S74	5.20E-07	96.55
KLPN.0918.00141.6_00003		KL64	Peptidase_S74	1.80E-07	91.38
KLPN.0918.00142.5_00012		KL64	Peptidase_S74	6.30E-07	91.38
KLPN.0918.00142.5_00017		KL64	Peptidase_S74	6.30E-07	91.38
KLPN.0918.00143.5_00056		KL64	Peptidase_S74	6.30E-07	91.38
KLPN.0918.00143.5_00062		KL64	Peptidase_S74	4.60E-07	91.38
KLPN.0918.00144.6_00080		KL64	Peptidase_S74	6.30E-07	91.38
KLPN.0918.00144.6_00085		KL64	Peptidase_S74	6.30E-07	91.38
KLPN.0918.00145.5_00087		KL64	Peptidase_S74	6.30E-07	91.38
KLPN.0918.00145.5_00094		KL64	Peptidase_S74	6.30E-07	91.38
KLPN.0918.00146.5_00053		KL64	Peptidase_S74	6.30E-07	91.38

KLPN.0918.00146.5_00058		KL64	Peptidase_S74	7.40E-08	91.38
KLPN.0918.00147.5_00023		KL64	Peptidase_S74	6.30E-07	91.38
KLPN.0918.00147.5_00028		KL64	Peptidase_S74	6.30E-07	91.38
KLPN.0918.00150.2_00001		KL30	Peptidase_S74	4.80E-07	96.55
KLPN.0918.00150.3_00096		KL30	Peptidase_S74	3.90E-09	93.10
KLPN.0918.00152.3_00062		KL10	Peptidase_S74	0.00067	93.10
KLPN.0918.00154.11_00048		KL107	Peptidase_S74	3.90E-09	93.10
KLPN.0918.00154.2_00033		KL107	Peptidase_S74	5.20E-07	96.55
KLPN.0918.00157.5_00061		KL64	Peptidase_S74	3.90E-09	93.10
KLPN.0918.00157.6_00097		KL64	Peptidase_S74	6.30E-07	91.38
KLPN.0918.00157.6_00103		KL64	Peptidase_S74	4.50E-08	91.38
KLPN.0918.00159.4_00002		KL21	Peptidase_S74	0.00012	93.10
KLPN.0918.00163.2_00002	#62	KL107	Peptidase_S74	4.80E-07	96.55
KLPN.0918.00164.8_00057		KL103	Peptidase_S74	5.50E-07	96.55
KLPN.0918.00165.2_00002		KL107	Peptidase_S74	4.80E-07	96.55
KLPN.0918.00166.6_00003		KL64	Peptidase_S74	1.80E-07	91.38
KLPN.0918.00167.7_00053		KL51	Peptidase_S74	3.90E-09	93.10
KLPN.0918.00168.5_00015		KL64	Peptidase_S74	3.70E-07	91.38
KLPN.0918.00168.5_00020		KL64	Peptidase_S74	6.30E-07	91.38
KLPN.0918.00181.2_00072		KL7	Peptidase_S74	3.90E-09	93.10
KLPN.0918.00183.5_00042		KL112	Peptidase_S74	3.90E-09	93.10
KLPN.0918.00189.4_00002		KL21	Peptidase_S74	0.00012	93.10
KLPN.0918.00191.4_00003	#47	KL124	Peptidase_S74	3.90E-09	93.10
KLPN.0918.00196.3_00005	#45	KL116	Peptidase_S74	3.90E-09	93.10
KLPN.0918.00197.1_00104	#54	KL2	Peptidase_S74	3.90E-09	93.10
KLQQ.0918.00001.2_00002	#36	KL35	Peptidase_S74	4.00E-06	91.38
KLQQ.0918.00002.2_00049	#41	KL125	Peptidase_S74	6.10E-07	93.10
KLQQ.0918.00005.3_00020	#49	KL138	Peptidase_S74	5.40E-07	96.55
KLQS.0918.00002.3_00002	#60	KL57	Peptidase_S74	0.00012	93.10
KLQS.0918.00005.1_00015	#29	KL111	Peptidase_S74	5.60E-07	96.55
KLQU.0918.00003.3_00001		KL153	Peptidase_S74	5.20E-07	96.55
KLVA.0918.00003.4_00002		KL16	Peptidase_S74	7.70E-05	93.10
KLVA.0918.00005.2_00002	#24	KL30	Peptidase_S74	4.50E-07	91.38
KLVA.0918.00006.4_00002		KL103	Peptidase_S74	0.0001	93.10
KLVA.0918.00006.7_00006		KL103	Peptidase_S74	4.10E-06	91.38
KLVA.0918.00007.2_00023		KL53	Peptidase_S74	1.20E-06	93.10
KLVA.0918.00008.2_00023		KL53	Peptidase_S74	1.20E-06	93.10
KLVA.0918.00009.2_00035		KL53	Peptidase_S74	1.20E-06	93.10
KLVA.0918.00010.1_00002	#35	KL31	Peptidase_S74	0.00031	93.10
KP06.0918.00001.2_00001	#39	KL51	Peptidase_S74	5.20E-07	96.55

Table S4. BLASTP protein hits from "intact" prophages against sequences of experimentally-validated capsule depolymerases. Hits were detected using *blastp*, and filtering by the e-value (maximum 1e-5), identity (40%) and coverage (40%).

Phage protein hit	Strain #	Host CLT	Profile	Identity (%)	Bitscore	Coverage (%)
KLOX.0918.00010.10_00052		KL70	YP_009226010.1	40.88	714	74.70
KLPN.0918.00168.5_00021		KL64	YP_009226010.1	41.09	714	74.70
KLPN.0918.00147.5_00029		KL64	YP_009226010.1	41.09	714	74.70
KLPN.0918.00146.5_00052		KL64	YP_009226010.1	41.09	714	74.70
KLPN.0918.00145.5_00086		KL64	YP_009226010.1	41.09	714	74.70
KLPN.0918.00144.6_00079		KL64	YP_009226010.1	41.09	714	74.70
KLPN.0918.00143.5_00055		KL64	YP_009226010.1	41.09	714	74.70
KLPN.0918.00142.5_00018		KL64	YP_009226010.1	41.09	714	74.70
KLPN.0918.00121.5_00018		KL64	YP_009226010.1	41.09	714	74.70
KLMI.0918.00007.3_00066		KL43	YP_009226010.1	41.09	714	74.70
KLPN.0918.00154.2_00032		KL107	YP_009226010.1	40.88	712	74.70
KLOX.0918.00010.2_00053		KL70	YP_009226010.1	42.87	712	68.61
KLPN.0918.00076.4_00013		KL27	YP_009226010.1	40.99	712	74.70
KLPN.0918.00034.2_00032		KL64	YP_009226010.1	41.20	712	74.70
KLPN.0918.00166.6_00004		KL64	YP_009226010.1	40.99	711	74.70
KLPN.0918.00141.6_00004		KL64	YP_009226010.1	40.99	711	74.70
KLPN.0918.00126.7_00007		KL64	YP_009226010.1	40.99	711	74.70
KLPN.0918.00102.5_00066		KL64	YP_009226010.1	40.99	711	74.70
KLPN.0918.00101.5_00024		KL64	YP_009226010.1	40.99	711	74.70
KLVA.0918.00009.2_00034		KL53	YP_009226010.1	40.99	711	74.70
KLVA.0918.00008.2_00022		KL53	YP_009226010.1	40.99	711	74.70
KLVA.0918.00007.2_00022		KL53	YP_009226010.1	40.99	711	74.70
KLVA.0918.00006.7_00007		KL103	YP_009226010.1	40.80	711	74.70
KLPN.0918.00150.2_00002		KL30	YP_009226010.1	40.06	711	77.21
KLQS.0918.00002.3_00003	#60	KL57	YP_009226010.1	40.88	711	74.70
KLPN.0918.00165.2_00003		KL107	YP_009226010.1	40.77	711	74.70
KLPN.0918.00163.2_00003	#62	KL107	YP_009226010.1	40.77	711	74.70
KLPN.0918.00152.3_00061		KL10	YP_009226010.1	41.09	711	74.70
KLPN.0918.00094.3_00003		KL1	YP_009226010.1	40.88	711	74.70
KLPN.0918.00057.11_00065		KL19	YP_009226010.1	40.88	711	74.70
KLPN.0918.00051.4_00026		KL30	YP_009226010.1	40.99	711	74.70

KLPN.0918.00050.2_00002		KL30	YP_009226010.1	40.99	711	74.70
KLPN.0918.00036.3_00003		KL1	YP_009226010.1	40.88	711	74.70
KLPN.0918.00032.1_00028		KL1	YP_009226010.1	40.88	711	74.70
KLPN.0918.00020.1_00029		KL1	YP_009226010.1	40.88	711	74.70
KLPN.0918.00012.2_00003		KL1	YP_009226010.1	40.88	711	74.70
KLPN.0918.00002.2_00002		KL1	YP_009226010.1	40.88	711	74.70
KLMI.0918.00001.2_00026		KL152	YP_009226010.1	40.88	711	74.70
KLPN.0918.00114.8_00082		KL64	YP_009226010.1	40.88	710	74.70
KLVA.0918.00003.4_00003		KL16	YP_009226010.1	40.88	709	74.70
KLPN.0918.00157.6_00096		KL64	YP_009226010.1	40.88	709	74.70
KLPN.0918.00133.4_00038		KL47	YP_009226010.1	40.77	709	74.70
KLPN.0918.00133.14_00038		KL47	YP_009226010.1	40.77	709	74.70
KLPN.0918.00189.4_00003		KL21	YP_009226010.1	40.67	708	74.70
KLPN.0918.00159.4_00003		KL21	YP_009226010.1	40.67	708	74.70
KLPN.0918.00135.4_00038		KL47	YP_009226010.1	40.77	708	74.70
KLPN.0918.00135.14_00038		KL47	YP_009226010.1	40.77	708	74.70
KLPN.0918.00132.4_00003		KL21	YP_009226010.1	40.67	708	74.70
KLPN.0918.00131.7_00002		KL64	YP_009226010.1	40.77	708	74.70
KLPN.0918.00069.6_00003		KL107	YP_009226010.1	40.77	708	74.70
KLPN.0918.00058.4_00003		KL21	YP_009226010.1	40.67	708	74.70
KLPN.0918.00033.2_00028		KL47	YP_009226010.1	40.77	708	74.70
KLPN.0918.00018.5_00002		KL47	YP_009226010.1	40.77	708	74.70
KLPN.0918.00017.3_00002		KL47	YP_009226010.1	40.77	708	74.70
KLQQ.0918.00005.3_00019	#49	KL138	YP_009226010.1	40.34	707	74.70
KLPN.0918.00028.1_00028		KL1	YP_009226010.1	40.77	707	74.70
KLQQ.0918.00001.2_00003	#36	KL35	YP_009226010.1	40.67	706	74.70
KLPN.0918.00057.7_00021		KL19	YP_009226010.1	40.45	706	74.70
KLPN.0918.00009.3_00026		KL67	YP_009226010.1	40.67	704	74.70
KLOX.0918.00007.5_00009		KL68	YP_009226010.1	42.59	704	68.13
KLOX.0918.00006.8_00050		KL68	YP_009226010.1	42.59	704	68.13
KLOX.0918.00005.8_00050		KL68	YP_009226010.1	42.59	704	68.13
KLAE.0918.00009.3_00006		KL107	YP_009226010.1	42.40	699	68.61
KLAE.0918.00007.7_00006		KL68	YP_009226010.1	42.40	699	68.61
KLPN.0918.00069.5_00004		KL107	YP_009226010.1	42.81	697	67.96

Table S5. Primers used in this study.

Name	Direction	Use	Sequence
Wza mutant construction			
KL1.wza.600-5	Forward	Construction of wza mutant in strain #56	TAGCTTCTATGGGCAGATGG
KL1.wza.600-3	Reverse	Construction of wza mutant in strain #56	CTGCTTCATATACGGGTGGAGC
KL1.wza.atg-3	Reverse	Construction of wza mutant in strain #56	AATGTCACATCATTAGTAAACC
KL1.wza.out-5	Forward	Verification of wza mutant in strain #56	GTTTCACCTTCACGCCATTCC
KL1.wza.out-3	Reverse	Verification of wza mutant in strain #56	TTTACTGCCGTCATCACCACG
KL30.wza.600-5	Forward	Construction of wza mutant in strain #24	ATGAACCGGGTAACCAACTGG
KL30.wza.600-3	Reverse	Construction of wza mutant in strain #24	TATCTCTGGTGCTGATTGCTGG
KL30.wza.L5	Forward	Construction of wza mutant in strain #24	GGCTAACACAGCTGCTCAGGAATTGGCAA ATTTTGGTTTACTGATGATGTGACATTGAC ATATGTTTGACTCTGTATTAGTGG
KL30.wza.atg-3	Reverse	Construction of wza mutant in strain #24	ATGCACATCATCAGTAAACC
KL30.wza.out-5	Forward	Verification of wza mutant in strain #24	TTTCACCTTCACGCCGTTCC
KL30.wza.out-3	Reverse	Verification of wza mutant in strain #24	ACCTTTAATCTCACCGGCAC
KL2.wza.500-5	Forward	Construction of wza mutant in strain #26	TTAACTGGTATGTGGAAGCGCATG
KL2.wza.500-3	Reverse	Construction of wza mutant in strain #26	TCACCAACCATTCGACCAAAG
KL2.wza.5	Forward	Construction of wza mutant in strain #26	ATGTTTAGTACAATATTAATTGTTTGC
KL2.wza.L3	Reverse	Construction of wza mutant in strain #26	GCGAACGGCATATATTCCCTGTGCAAACA ATTAATATTGTACTAAACATAATGTCACA TCATCAGTAAATCAAAATTTGC
KL2.wza.out-5	Forward	Verification of wza mutant in #26	ACCGGGACAGATAACGAACC
KL2.wza.out-3	Reverse	Verification of wza mutant in #26	CACTTAACCTTGCCCATCCACG
WcaJ mutant construction			

KL2.wcaJ.L5	Forward	Verification of wcaJ mutant in #57 and #58	gttacgttaatgaaacttatcgacagatatgcttgtttataaatgagtgattgaattctagatgctccttaagacaagg
KL2.wcaJ.500-3	Reverse	Verification of wcaJ mutant in #57 and #58	CACCATACTCAATGCCGTTATGC
KL2.wcaJ.L3	Reverse	Verification of wcaJ mutant in #57 and #58	aattcaatcactcatttataaac
KL2.wcaJ.500-5	Forward	Verification of wcaJ mutant in #57 and #58	ttcttaaacttaagaacataagagc
KL2.wcaJ.out-5	Forward	Verification of wcaJ mutant in #57 and #58	GAATGGAATTGTTCTGCCTCTGAC
KL2.wcaJ.out-3	Reverse	Verification of wcaJ mutant in #57 and #58	CTCAGTTCACCTTCGTTCCATTCG
Phage detection & recircularization			
54_Ph01-R_circ	Reverse	Tests for recircularisation of Prophage 1 in strain #54	GGTGATCGCTTCCTGTGACGTGTTTTCC
54_Ph01-F1_circ	Forward	Tests for recircularisation of Prophage 1 in strain #54	GGCGTGTTCTGGATTTCTACC
54_Ph01-F2_circ	Forward	Tests for recircularisation of Prophage 1 in strain #54.	TAATGAACCAGTGCCCGTCTCTGCCTTCC
54_Ph03_F	Forward	Tests for recircularisation of Prophage 3 in strain #54	CGAGATGGTACGCCGTTATG
54_Ph03_R	Reverse	Tests for integrase, predicted 120bp (in genome)	GATTTCCTCCCTGTGTCGTATT
54_Ph03_recirc	Forward	Tests for recircularisation of Prophage 3 in strain #54	TTTCTCAAACAGCGGGATGG
54_Ph01_R2	Reverse	Tests for presence of Prophage 1 of strain #54.	GCCGATTTGTTGCGAGATCC
54_Ph03_F2	Forward	Tests for presence of Prophage 3 of strain #54.	GCGTCTGACAAACCGATAACAGC
54_Ph03_R2	Reverse	Tests for presence of Prophage 3 of strain #54.	ACGGCGTAAAGAAATGACAGTGCC
54_Ph02_F	Forward	Tests for presence of Prophage 2 of strain #54.	AAGCCTACAAACTGACTGACG
54_Ph02_R	Reverse	Tests for presence of Prophage 2 of strain #54.	TGACAAAGGAACAAGGTGAGG
54_Ph02_Fcirc	Forward	Tests for recircularization of Prophage 2 of strain #54.	CGATACCTCTGACGCTCTGG
54_Ph04_F	Forward	Tests for presence of Prophage 4 of strain #54.	GAAGGTAAGTTTGTCGGCCAGC
54_Ph04_R	Reverse	Tests for presence of Prophage 4 of strain #54.	TCATCCGGCACATCTTCGAGG
54_Ph04_Fcirc	Forward	Tests for recircularization of Prophage 4 of strain #54.	ATCGTTGTCAGTATCGGTGG
54_Ph01_R3	Reverse	Tests for recircularization of Prophage 3 of strain #54.	CTCAACGCCTGGGCTATTGC
25_Ph01_F	Forward	Tests for presence (and recircularization) of Prophage 1 of strain #25.	GAATGCTGTATCCGGTGGTTGC

25_Ph01_R	Reverse	Tests for presence of Prophage 1 of strain #25.	GTGCATGTTCGGTCAGGTGG
25_Ph01_R_circ	Reverse	Tests for recircularisation of Prophage 1 of strain #25	AACGGCTTCGTCGTACATCG
36_Ph01_F	Forward	Tests for presence of Prophage 1 of strain #36.	CGGTAGTTCGATGCCTGTTCTTTCC
36_Ph02_F	Forward	Tests for presence of Prophage 2 of strain #36.	CTGTACCACGGTCACCAAATCG
36_Ph02_R	Reverse	Tests for presence of Prophage 2 of strain #36.	ACCGCCGATTACACAACACG
36_Ph02_Fcirc	Forward	Tests for recircularization of Prophage 2 of strain #36.	GGCAACAACACGCGGATCTCC
36_Ph03_F	Forward	Tests for presence of Prophage 3 of strain #36.	ATATCAGTGATCGACCGGGAGC
36_Ph03_R	Reverse	Tests for presence of Prophage 3 of strain #36.	ATAAACTGTCTGATGGCGGTGG
36_Ph03_Rcirc	Reverse	Tests for recircularization of Prophage 3 of strain #36.	GATGAGGGCTTTATTGTAGGTGG
36_Ph04_F	Forward	Tests for presence of Prophage 4 of strain #36.	AAGGATTTCTCCTGCCACACC
36_Ph04_R	Reverse	Tests for presence of Prophage 4 of strain #36.	GAATCCTTCTTTGCGCGTCG
36_Ph04_Rcirc	Reverse	Tests for recircularization of Prophage 4 of strain #36.	GGGATACAAACCAACCTGACG
36_Ph03_R2	Reverse	Tests for recircularization of Prophage 3 of strain #36.	GTATACCCTGAAGTCTCGCTGG
36_Ph04_R2	Reverse	Tests for recircularization of Prophage 4 of strain #36.	GTGCGGTCAGCAAATACTGG
37_Ph01_F	Forward	Tests for presence of Prophage 1 of strain #37.	AGGAAAGGATTTCTCTGAGAGC
37_Ph01_R	Reverse	Tests for presence of Prophage 1 of strain #37.	GGCTATCCTGAACGAACTCAATCG
37_Ph01_Fcirc	Forward	Tests for recircularization of Prophage 1 of strain #37.	ACCGATCTTCTCTACCCAGC
37_Ph02_F	Forward	Tests for presence of Prophage 2 of strain #37.	CTAACGCATCCTGCAACTGATTTCC
37_Ph02_R	Reverse	Tests for presence of Prophage 2 of strain #37.	GGCGGAATTGGTCTCGTTGC
37_Ph02_Fcirc	Forward	Tests for recircularization of Prophage 2 of strain #37.	GCAAAGCCCAACCTGACACC
37_Ph01_Fc2	Forward	Tests for recircularization of Prophage 1 of strain #37.	ATCATGGAGGGTCAGCTGCTGG
37_Ph02_Fc2	Forward	Tests for recircularization of Prophage 2 of strain #37.	TGTTCATCATGGTTTGGGTGACG
37_Ph02_refFc	Forward	Tests for recircularization of Prophage 2 of strain #37	TAAAGCGTTCTCAGGTTTCCTCGGG
37_Ph02_refRc	Reverse	Tests for recircularization of Prophage 2 of strain #37	TGGCTGGTAACGTGATCTTAATGC
46_Ph01_F	Forward	Tests for presence of Prophage of strain #46.	TGGGCTCATCAGCATTGAAGG
46_Ph01_R	Reverse	Tests for presence of Prophage of strain #46.	CCATGTTCACGCCAGTGTCG
46_Ph01_Fcirc	Forward	Tests for recircularization of Prophage of strain #46.	TTGCCAGAATGGCCTGATTTCG

46_Ph01_Fc2	Forward	Tests for recircularization of Prophage 1 of strain #46	TACAGGAAGGATACGGATTTCAGC
48_Ph01_F	Forward	Tests for presence of Prophage of strain #48.	ACAGGTGAGCGTAGACCATCG
48_Ph01_R	Reverse	Tests for presence of Prophage of strain #48.	ATTCTCTGTTGGTTGCCCTGC
48_Ph01_F_circ	Forward	Tests for recircularization of Prophage of strain #48.	CTCATACCTGGGTTGCTTGC
48_Ph01_R_bis	Reverse	Tests for recircularization of Prophage 1 of strain #48	TTCTGGAGCGGGAAGAGATCAGC
50_Ph03_Fc	Forward	Tests for recircularization of Prophage 3 of strain #50	CTTACTTTCGGGCCATGTTCAACG
50_Ph03_R	Reverse	Tests for presence of Prophage 3 of strain #50	CGGATTTACGACCAGCATCGAAC
50_Ph03_F	Forward	Tests for presence of Prophage 3 of strain #50	GATATGGCAGACCAGAGGTTACG
51_Ph01_Fc	Forward	Tests for recircularization of Prophage 1 of strain #51	GCGTACTGGCTGGATCTTAATTTCG
51_Ph01_R	Reverse	Tests for presence of Prophage 1 of strain #51	CGAGAAGCATACCCAGTTCAACC
51_Ph01_F	Forward	Tests for presence of Prophage 1 of strain #51	GCCTTACCAAATAACAACCCTCG
55_Ph01_refFc	Forward	Tests for recircularization of Prophage 2 of strain #55	GGATTTCTGCGGTTACGTTGTGC
55_Ph01_refR	Reverse	Tests for presence of Prophage 1 of strain #55	GCACTGTCATTTCTTTACGCCGTC
55_Ph01_refF	Forward	Tests for presence of Prophage 1 of strain #55.	CTCTCCCATCCTCCCATTTCCTG
62_Ph01_F	Forward	Tests for presence of Prophage 1 of strain #62.	AAGCCAGGGATGAACTGATAGC
62_Ph01_R	Reverse	Tests for presence of Prophage 1 of strain #62.	ACAGCACACCGATTTCTTCC
62_Ph01_Fcirc	Forward	Tests for recircularization of Prophage 1 of strain #62.	AGCAACAGCTCGCAAATCTCC
62_Ph02_F	Forward	Tests for presence of Prophage 2 of strain #62.	ACGGTTAAACAGCGAGAAGC
62_Ph02_R	Reverse	Tests for presence of Prophage 2 of strain #62.	CAGTATCACCAGTACCCAGC
62_Ph02_F circ	Forward	Tests for recircularization of Prophage 2 of strain #62.	AGTCCTTTCCACTGCTTACC
62_Ph02_Fcirc_bis	Forward	Tests for recircularization of Prophage 2 of strain #62.	TGCCAGTTTGTTTGCTTTCGTCC

Table S6. pVOGS associated with phage repressors.

pVOG
VOG0523
VOG4990
VOG4613
VOG0126
VOG4543
VOG9234
VOG0988
VOG1681
VOG2312
VOG7724
VOG0286
VOG5346
VOG9725
VOG0513
VOG8287
VOG4597
VOG6627
VOG11077
VOG8296
VOG4696
VOG4908
VOG2167
VOG9374
VOG4614
VOG5206
VOG1658
VOG0743
VOG6362

SUPPLEMENTAL REFERENCES

Lin TL, Hsieh PF, Huang YT, Lee WC, Tsai YT, Su PA *et al* (2014). Isolation of a bacteriophage and its depolymerase specific for K1 capsule of Klebsiella pneumoniae: implication in typing and treatment. *J Infect Dis* **210:** 1734-1744.

Majkowska-Skrobek G, Latka A, Berisio R, Maciejewska B, Squeglia F, Romano M *et al* (2016). Capsule-Targeting Depolymerase, Derived from Klebsiella KP36 Phage, as a Tool for the Development of Anti-Virulent Strategy. *Viruses* **8:** 324.

Pan YJ, Lin TL, Lin YT, Su PA, Chen CT, Hsieh PF *et al* (2015). Identification of capsular types in carbapenem-resistant *Klebsiella pneumoniae* strains by *wzc* sequencing and implications for capsule depolymerase treatment. *Antimicrob Agents Chemother* **59:** 1038-1047.

Pan YJ, Lin TL, Chen CC, Tsai YT, Cheng YH, Chen YY *et al* (2017). Klebsiella Phage PhiK64-1 Encodes Multiple Depolymerases for Multiple Host Capsular Types. *J Virol* **91:** e02457-02416.
